# Deciphering the clinico-radiological heterogeneity of dysexecutive Alzheimer’s disease

**DOI:** 10.1093/cercor/bhad017

**Published:** 2023-01-31

**Authors:** Nick Corriveau-Lecavalier, Leland R Barnard, Jeyeon Lee, Ellen Dicks, Hugo Botha, Jonathan Graff-Radford, Mary M Machulda, Bradley F Boeve, David S Knopman, Val J Lowe, Ronald C Petersen, Clifford R Jack, Jr, David T Jones

**Affiliations:** Department of Neurology, Mayo Clinic, Rochester, MN 55905, USA; Department of Neurology, Mayo Clinic, Rochester, MN 55905, USA; Department of Radiology, Mayo Clinic, Rochester, MN 55905, USA; Department of Neurology, Mayo Clinic, Rochester, MN 55905, USA; Department of Neurology, Mayo Clinic, Rochester, MN 55905, USA; Department of Neurology, Mayo Clinic, Rochester, MN 55905, USA; Department of Psychiatry and Psychology, Mayo Clinic, Rochester, MN 55905, USA; Department of Neurology, Mayo Clinic, Rochester, MN 55905, USA; Department of Neurology, Mayo Clinic, Rochester, MN 55905, USA; Department of Radiology, Mayo Clinic, Rochester, MN 55905, USA; Department of Neurology, Mayo Clinic, Rochester, MN 55905, USA; Department of Radiology, Mayo Clinic, Rochester, MN 55905, USA; Department of Neurology, Mayo Clinic, Rochester, MN 55905, USA; Department of Radiology, Mayo Clinic, Rochester, MN 55905, USA

**Keywords:** dysexecutive Alzheimer’s disease, FDG-PET, behavioral neurology, machine learning, neuropsychology

## Abstract

Dysexecutive Alzheimer’s disease (dAD) manifests as a progressive dysexecutive syndrome without prominent behavioral features, and previous studies suggest clinico-radiological heterogeneity within this syndrome. We uncovered this heterogeneity using unsupervised machine learning in 52 dAD patients with multimodal imaging and cognitive data. A spectral decomposition of covariance between FDG-PET images yielded six latent factors (“eigenbrains”) accounting for 48% of variance in patterns of hypometabolism. These eigenbrains differentially related to age at onset, clinical severity, and cognitive performance. A hierarchical clustering on the eigenvalues of these eigenbrains yielded four dAD subtypes, i.e. “left-dominant,” “right-dominant,” “bi-parietal-dominant,” and “heteromodal-diffuse.” Patterns of FDG-PET hypometabolism overlapped with those of tau-PET distribution and MRI neurodegeneration for each subtype, whereas patterns of amyloid deposition were similar across subtypes. Subtypes differed in age at onset and clinical severity where the heteromodal-diffuse exhibited a worse clinical picture, and the bi-parietal had a milder clinical presentation. We propose a conceptual framework of executive components based on the clinico-radiological associations observed in dAD. We demonstrate that patients with dAD, despite sharing core clinical features, are diagnosed with variability in their clinical and neuroimaging profiles. Our findings support the use of data-driven approaches to delineate brain–behavior relationships relevant to clinical practice and disease physiology.

## Introduction

Dysexecutive Alzheimer’s disease (dAD) is a recently described clinical phenotype of young-onset Alzheimer’s disease initially presenting as a progressive and predominant degradation of core executive functions in the absence of prominent behavioral features (Townley et al. 2020). In addition to a younger age of onset, this variant of Alzheimer’s disease generally harbors higher levels of neocortical tau pathology, has a shorter disease duration, and lacks the predominant amnestic profile compared to the canonical, amnestic variant of Alzheimer’s disease (Barnes et al. 2018; Ferreira et al. 2020; Smirnov et al. 2021a, 2021b). We recently published a clinical case series suggesting that dAD can be further divided in phenotypic subtypes based on their pattern of parieto-frontal network degeneration (Corriveau-Lecavalier et al. 2022a), which is the principal macro-scale underpinning of executive functions (Selemon and Goldman-Rakic 1988; Binkofski et al. 1999; Murphy et al. 2020). This yielded three phenotypic subtypes—“left-dominant,” “right-dominant,” and “bi-parietal-dominant.” These differed in their clinical and cognitive profiles, where left-dominant patients showed more pronounced impairment in verbal capacities, right-dominant patients were more impaired on tasks tapping into the processing of visual information, and bi-parietal patients generally had a milder cognitive impairment profile which was relatively restricted to cognitive flexibility (Corriveau-Lecavalier et al. 2022a). This suggests that individuals with dAD can present with a preferential impairment of variable aspects of executive functions, which is reflected in distinct subtypes. However, this study relied on coarse clinical observations in a handful of patients. In the present study, we aimed to extend this work by leveraging a larger dAD patient cohort and data-driven techniques best suited to further elucidate the clinico-radiological heterogeneity within this syndrome.

Methods traditionally used to assess the clinical and biological variability of neurological disorders usually consist of group-wise comparisons with clinical characterization or an a priori anatomical location as a starting point, an approach known as “forward inference.” Although this approach has provided a great deal of insight into the complexity of neurodegenerative diseases so far (Crutch et al. 2017; Buciuc et al. 2021), it also entails non-trivial limitations as it generally focuses on variability at the group rather than individual level, and is prone to biases from the observer. Recent years have seen an increased use of unsupervised machine learning techniques applied to brain imaging data to extricate the clinico-radiological variability of neurodegenerative diseases (Groot et al. 2020; Corriveau-Lecavalier et al. 2021; Levin et al. 2021; Townley et al. 2021; Jones et al. 2022). These methods have advantages over traditional methodologies, in that they exploit the inter-individual variability in pathophysiologically relevant brain data as a starting point to yield data-driven, unbiased latent variables that can subsequently be linked to clinical and cognitive features, an approach known as “reverse inference” (Seghier and Price 2018). Techniques such as spectral decomposition of covariance consist of the reduction of highly dimensional data (e.g. brain images) into a smaller set of plausible and biologically interpretable latent variables, which refer to gradients of macro-scale cortical organization in this particular context (Margulies et al. 2016; Seghier and Price 2018; Raut et al. 2020; Jones et al. 2022). A recent study pioneering this approach in normal aging and degenerative dementia disorders revealed that seven dementia syndromes including phenotypic spectrum of AD (typical AD, dAD, posterior cortical atrophy or PCA, logopenic variant of primary progressive aphasia or lvPPA) could be indexed by a low-dimensional latent space derived from ^18^fluorodeoxyglucose (FDG)-positron emission tomography (PET) images of a cohort composed of patients with AD (Jones et al. 2022). Other studies have used such methods to uncover the clinico-radiological heterogeneity within a single dementia syndrome (Groot et al. 2020; Townley et al. 2021). For instance, Townley et al. (2021) found that eight latent factors accounted for approximatively 50% of covariance in patterns of FDG-PET hypometabolism of 91 patients with PCA. Importantly, these eigenbrains differentially related to clinical and cognitive symptomatology, suggesting that these patterns of macro-scale network degeneration are tied to syndromic heterogeneity.

Unsupervised machine learning techniques have also been used to yield data-driven, unbiased disease subtyping solutions. A recent study by Levin et al. (2021) used a hierarchical clustering algorithm on FDG-PET images of patients from the ADNI cohort to derive three AD subtypes, a “limbic-dominant,” a “typical,” and a “cortical-dominant” subtype. These three subtypes, in addition to a “posterior” subtype, were also found in another study applying a clustering algorithm to tau-PET images of a large AD cohort, and each of these subtypes was associated with a distinct clinical profile and trajectory (Vogel et al. 2021). These clustering techniques may thus represent a promising alternative to classical approaches to delineate data-driven dAD subtypes.

Overall, data-driven approaches have the potential to delineate brain–behavior relationships and uncover disease subtypes. This could inform disease models and consequently contribute to clinical decision-making and therapeutics strategies aimed at disease biology. In this study, we aimed to decipher the clinico-radiological heterogeneity specific to dAD by using unsupervised machine learning techniques. In a first step, we performed a spectral decomposition of covariance between FDG-PET images in a cohort of dAD patients to yield latent patterns of macro-scale cortical network degeneration, and subsequently assessed relationships between these eigenbrains and clinical and cognitive features. In a second step, we applied a hierarchical spectral clustering using an affinity propagation algorithm on the eigenvalues of the FDG-PET-based eigenbrains to derive unbiased, data-driven dAD subtypes. We then compared imaging profiles (FDG-PET, amyloid-PET, tau-PET, magnetic resonance imaging or MRI) of each subtype to those of cognitively unimpaired and amyloid-negative controls, and compared demographic, clinical, and cognitive data between dAD subtypes.

## Materials and methods

### Participants

#### dAD cohort

Patients with dAD were seen in our tertiary behavioral neurology clinic at Mayo Clinic Rochester. They all met criteria for dAD (Townley et al. 2020) in that they presented with a predominant and progressive dysexecutive syndrome for at least 6 months, had positive cerebrospinal fluid (CSF) or PET biomarkers for Alzheimer’s disease pathophysiology or definite Alzheimer’s disease upon post-mortem evaluation (see criteria below), and did not have a medical condition better accounting for the clinical presentation. All patients underwent a clinical visit with an experienced neurologist subspecialized in behavioral neurology, which included an interview with the patient and an informant, a neurological examination, and an extensive review of relevant clinical documentation. A progressive dysexecutive syndrome was defined based on recently published criteria (Townley et al. 2020). This requires the presence of an insidious, continuous, and persistent decline in mental functions with the defining feature being that of a predominance of clinical symptoms originating from executive dysfunction. Meeting criteria for behavioral variant frontotemporal dementia (bvFTD; Rascovsky et al. 2011) is exclusionary, but also having behavioral symptoms that do not meet bvFTD criteria or co-existing memory, language, and/or visual symptoms is not exclusionary if the predominant symptoms are determined to be originating from an impairment in any core executive function (i.e. working memory, cognitive flexibility, and/or inhibition). This determination must be made by an experienced clinician with expertise in assessing the clinical implications of impairment in these cognitive domains using structured clinical interviews, physical examination, and formal neuropsychological testing in a similar fashion as the core criteria for primary progressive aphasia (PPA; Mesulam 2001) or mild cognitive impairment (Petersen et al. 2001). None of these criteria specify particular cognitive testing results that must be present, but such information can be used by the clinician in making the clinical diagnosis of all of these clinical syndromes. Construct validity for defining this clinical syndrome in this way has been demonstrated in the original report (Townley et al. 2020), and recently via mapping of this clinical syndrome to brain anatomy associated with executive function as opposed to memory, visual, or language functions as is the case for other AD associated clinical syndromes predominantly affecting those domains (Jones et al. 2022). Given the recent characterization of dAD (Townley et al. 2020), 21 patients were initially attributed a diagnosis of “early-onset AD dementia” and were later attributed a diagnosis of dAD based on retrospective review of medical records and consensus opinion from four behavioral neurologists. Of note, 45/52 patients included in this report came from the Townley et al.’s (2020) study. This study met HIPAA privacy standards and was approved by the Mayo Clinic Institutional Review Board. Patients and/or their designee provided written consent upon their clinical visit for their data to be used for research purposes. A subset of the patients (33/52) was subsequently enrolled in the Alzheimer’s disease Research Center (ADRC) protocol following their clinical visit, allowing for the collection of neuropsychological, MRI, amyloid-PET, and tau-PET imaging data. Patients coming from clinical practice and those enrolled in the ADRC protocol did not differ in terms of age, sex, or education (all *P*s > 0.4).

#### Controls participants

We collected data on 52 cognitively unimpaired controls enrolled in the ADRC or Mayo Clinic Study of Aging who exactly matched the 52 dAD patients on age and sex and were selected according to FDG-PET and amyloid-PET availability. All control participants had to be amyloid-negative based on PET imaging to be included in the study, and those with available tau-PET also had to be tau-negative.

### Alzheimer’s disease biomarkers assessment

#### Fluid biomarkers

Alzheimer’s disease pathophysiology was confirmed through CSF in 24/52 dAD patients over the course of clinical care. The fluid biomarkers collection procedure is thoroughly described in Townley et al. (2020) and was performed within a week from the clinical visit. CSF analysis was performed by Athena Diagnostic (Worcester, MA), which provides Aβ_42_, T-tau, and P-tau levels. An Aβ42-tau index (or ATI) is also provided, which has been shown to accurately distinguish clinically diagnosed AD from vascular dementia and bvFTD with respective sensitivity and specificity of 85–94 and 83–89% (Hulstaert et al. 1999; Andreasen et al. 2001). Alzheimer’s disease biomarker positivity cut-offs were the following: not consistent with Alzheimer’s disease (P-tau < 54 pg/ml; ATI > 1.2); borderline Alzheimer’s disease (P-tau 54–58 pg/ml; ATI 0.8–1.2); consistent with AD (P-tau > 58 pg/ml; ATI < 0.8). Borderline CSF ATI is often seen in dAD (Townley et al. 2020), with 24% of cases having low amyloid but normal P-tau levels.

#### Amyloid-PET and tau-PET

Amyloid-PET and tau-PET were acquired in research settings. Amyloid-PET was obtained for 27/52 dAD and all controls, and tau-PET was obtained for 25/52 dAD and 23/52 controls. Amyloid-PET and tau-PET images were acquired using Pittsburgh compound B (PiB) and ^18^F-AV-1451 ligands, respectively. Acquisition protocols and processing pipelines for PET images are described in separate publications (Jack et al. 2012, 2017). Briefly, amyloid-PET and tau-PET images were co-registered to their corresponding MRI image, normalized into the Mayo Clinic Adult Lifespan Template (MCALT) (available at https://www.nitrc.org/projects/mcalt/), and smoothed with a 6-mm full width at half-maximum using Statistical Parametric Mapping 12 (SPM12). Both amyloid-PET and tau-PET images were normalized to the cerebellar crus region. A global standardized uptake value ratio (SUVR) was yielded from a validated meta-region of interest for each patient (Jack et al. 2017). Thresholds for amyloid-PET and tau-PET positivity were set at > 1.42 and > 1.23, respectively (Jack et al. 2017). There was no statistical difference in age, education, or sex between the subset of controls and dAD patients with available PET imaging.

#### Post-mortem assessment

Alzheimer’s disease pathology was confirmed through post-mortem examination in one patient. The immunohistochemical examination protocol is described in Townley et al. (2020). Briefly, the presence of amyloid plaques and neurofibrillary tangles was assessed in the left hemisphere using antibodies to Aβ and P-tau and staged in accordance with the National Institute of Aging-Alzheimer’s Association (NIA-AA) and Consortium to Establish a Registry for Alzheimer’s Disease guidelines (Mirra et al. 1991; Hyman et al. 2012) (i.e. “ABC score”). Thal amyloid phase (Thal et al. 2002) and Braak tangle stages (Braak and Braak 1991) were performed using Aβ and tau immunochemistry, respectively.

#### FDG-PET

FDG-PET images were obtained in clinical settings for all patients and in research settings for all controls. These were acquired using a PET/CT scanner (GE healthcare) following a 30-minutes uptake period while waiting in a dimly lit room. The scanning session lasted 8 minutes which was split into four 2-minute dynamic frames following a low-dose CT transmission scan. Images were preprocessed using an MRI-free pipeline that consists of the registration of the FDG-PET image to the MCALT space using a non-linear symmetric diffeomorphic registration. Spatially normalized FDG-PET images were intensity normalized to the pons to produce SUVR images.

#### Magnetic resonance imaging

MRIs were obtained in research settings and were available for 33/52 dAD patients and all controls. MRI scans were acquired with a 3 T General Electronics (GE) scanner using a magnetization prepared rapid gradient echo sequence. Acquisition parameters were the following: repetition time = 2300 ms, echo time = 3 ms, T1 = 900 ms, flip angle of 8^o^, field of view = 26 cm, 256 × 256 in-plane matrix with a phase field of 0.94; slice thickness = 1.2 mm. Images were segmented using Unified Segmentation in SPM12 with MCALT population-optimized priors and settings, and MCALT atlases were normalized to each scan using ANTs. A smoothing of 6-mm full width at half-maximum was applied to resulting images using SPM12.

#### Cognitive assessment

Bedside cognitive screening was performed with the Short Test of Mental Status (STMS; Kokmen et al. 1991) for all dAD patients during neurological examination. Neuropsychological assessment was performed either in clinical or research settings for 35/52 dAD patients. Test selection was not standardized and varied as a function of the setting in which it was performed and/or degree of cognitive impairment.

Raw scores were transformed into age-adjusted scaled scores. Scaled scores for the WAIS, WMS, and D-KEFS were calculated using their respective standard manuals. Scaled scores for the remaining tests were calculated using the Mayo Older Americans Normative Studies (MOANS; Petersen et al. 1992; Lucas et al. 1998; Steinberg et al. 2005; Machulda et al. 2007). As MOANS are only available for individuals of age 56 and older, the youngest age bracket (56–60) was used for patients younger than 56. Discontinued performance was assigned a scale score of 1.

Assessments included a combination of tests and covered cognitive domains including cognitive flexibility (Trail making test B or TMT-B; Spreen and Strauss 1998), Wisconsin Card Sorting Test or WCST (Grant and Berg 1993), inhibition (Stroop inhibition; Stroop 1935), working memory (WAIS-III/IV: digit span, arithmetic, letter-number sequencing; Wechsler 1997, 2008), verbal episodic memory (Rey Auditory Verbal Learning Test or RAVLT; Rey 1964), visual episodic memory (WMS-III Visual Reproduction I and II; Weschler 1987), verbal fluency (animal and phonemic fluency; Tombaugh et al. 1999), visuoconstruction (WAIS-III/IV: block design; Wechsler 1997, 2008), Rey-Osterrieth Complex Figure copy or ROCF (Osterrieth 1944), and visuospatial reasoning (WAIS-III/WAIS-IV: picture completion, matrix reasoning, visual puzzles; Wechsler 1997, 2008). As neuropsychological assessment was not standardized across patients, composite scores were computed for each cognitive domain by averaging scaled scores of tests included in each of these cognitive domains. Scores on the WCST and Trail TMT-B were considered separately.

#### Spectral decomposition of covariance analysis

We performed a spectral decomposition of covariance using a principal component analysis on FDG-PET images of dAD patients. This was done in Python version 3.7.12 using libraries developed in-house. The aim of this analysis was to provide a biologically interpretable low-dimensional latent space expressing inter-individual variability in patterns of macro-scale cortical metabolism (see Jones et al. 2022 for more details). First, pons-normalized FDG-PET images were median-centered at zero and scaled by their interquartile range (IQR) and censored with a brain tissue mask. The 3D brain volume of each image was then flattened into a 1D array of voxels and entered into a subject-by-voxel matrix. This high-dimensionality matrix was then submitted to a singular value decomposition to derive a set of latent factors. These factors are referred to as “eigenbrains,” and their values take the same dimensions of the masked template space. These eigenbrains are represented by gradients of metabolism organized along dimensions determined by their spatial distribution and magnitude of intensity. It is important to bear in mind that an eigenbrain does not reflect hypometabolism per se, but rather a relative distribution of metabolism across the entire brain with opposing poles of relative hypo- and hyper-metabolism. Hence, a patient could show less metabolism in a set of regions compared with another set of regions reflected by a given eigenbrain, and another patient could show the exact opposite pattern. This directionality of this patient-level pattern is determined by the loading factor on a given eigenbrain, referred as to an “eigenvalue.” This eigenvalue can be either positive or negative and describes how the pattern of hypometabolism in a given patient relates to the topology and directionality of a given eigenbrain. These individual eigenvalues can then be subsequently used as predictors of variables of interest (e.g. demographic, clinical, cognitive) to determine how each eigenbrain related to these variables at the group-level (this is described in more detail in the Statistical analyses section). Of note, the directionality of the eigenvalues is arbitrary and has no meaning per se. This means that the directionality of the eigenvalues could be flipped for each eigenbrain, and the interpretation of the brain–behavior relationships would remain the same. However, the association between eigenvalues and cognition are meaningful within the context of the directionality of the eigenvalues once determined.

Each eigenbrain accounts for a proportion of covariance in FDG-PET patterns in a descending order. We determined the number of eigenbrain to be retained for further analysis using Horn’s parallel analysis (Horn 1965). Briefly, this method is rooted in the sampling theory and proposes that factoring should cease when factors cannot account for a proportion of variance that is higher than expected by chance. This was determined using the “latent-root criterion,” which is based on the comparison of latent roots of each eigenbrain to those of random variables with identical dimensions, which are all equal to 1. Thus, eigenbrains with a latent root greater than one were retained for further analysis.

#### Meta-analytic functional decoding

A meta-analytic decoding of the eigenbrains was performed using the *Neurosynth* database (Poldrack et al. 2012; Rubin et al. 2017), which groups activation maps of approximatively 14,000 functional neuroimaging studies into meta-analytic topics reflecting a wide range of cognitive and behavioral functions. We used the 50 topics list (version 7; https://neurosynth.org/analyses/topics/). Consistent with studies using this approach (Margulies et al. 2016; Shine et al. 2021; Jones et al. 2022), we removed 28 topics because they did not capture coherent cognitive or behavioral functions. Topics retained for analysis are listed in Supplementary Table 1. This procedure allows for the identification of topics that best align with the spatial and directional pattern (i.e. positive and negative loadings weights) of each eigenbrain. This allows for an interpretation of plausible brain–behavior relationships associated with each eigenbrain by leveraging a large body of functional neuroimaging studies. Of note, interpretation should be guided by the directionality and relative strength of associations between eigenbrains and meta-analytic topics rather than absolute coefficients values, for reasons cited on the *Neurosynth* website (https://neurosynth.org/faq/#q16). It is important to keep in mind that this analysis is meant to be descriptive, and no statistical significance testing is performed.

#### Hierarchical spectral clustering

We performed a hierarchical spectral clustering analysis on eigenvalues of significant FDG-PET-based eigenbrains to derive data-driven dAD subtypes. This was done using the *R* apcluster package (https://cran.r-project.org/web/packages/apcluster/; Bodenhofer et al. 2011). We first created a patient-by-patient similarity matrix using the negative squared distance between scaled eigenvalues for each significant eigenbrain. This matrix was submitted to an affinity propagation clustering algorithm, which is based on the identification of “exemplars” data points and the formation of clusters around these points (Frey and Dueck 2007). The exemplars and their cluster members are determined through an iterative process aiming to optimize within- and between-cluster distance. Multiple distance metrics were compared, including the Euclidean, Manhattan, and correlation-based distances. The optimal distance metric was determined based on silhouette coefficient values (Rousseeuw 1987), which express how similar a data point is to its own cluster compared with other clusters, where a higher value indicates a better clustering solution. We applied a 10% quantile of similarities threshold to avoid clustering solutions with elevated numbers of clusters with small number of patients.

### Statistical analyses

Analyses were performed using a mix of *R* (https://www.r-project.org/), Python 3.7 (https://www.python.org/downloads/release/python-370/), and Matlab https://www.mathworks.com/products/matlab.html). We first used a regression framework to assess the relationship between eigenbrains and demographic (age at symptom onset, education), clinical (STMS), and cognitive (cognitive domains) variables. For each demographic and cognitive variable, eigenvalues of each eigenbrain were entered as predictors in a multivariable regression model. This allowed to determine which eigenbrain(s) significantly and independently predicted the variable of interest.

We then performed pair-wise comparisons between voxel-wise patterns of FDG-PET metabolism of each dAD subtype derived from the hierarchical clustering analysis to those of the control group. This consisted in calculating the mean and standard deviation of each voxel of the brain for each group (controls and each dAD subtype), and then calculating the *Z* score of each voxel in a given patient group relative to the mean image of the control group, resulting in voxel-wise *Z* score maps. These comparisons were repeated for amyloid-PET, tau-PET, and MRI images to determine the extent to which patterns of neurodegeneration, tau deposition, and amyloid deposition overlapped with those of FDG-PET hypometabolism. We additionally produced patient-level *Z* score maps, in which we fitted “prototypical” patients of each dAD subtype to the control group (Figs 2 and 4). We then compared demographic, clinical, and cognitive data between dAD subtypes using ANOVAs with Tukey’s test for post-hoc comparisons for continuous variables and chi square analyses for categorical variables.

## Results

### Demographic data and AD biomarkers

Demographic, clinical, cognitive, and AD biomarkers data for the dAD cohort are summarized in Table 1. There were 34 females and 18 males. The average age at symptom onset was 53.44, whereas the average age at clinical presentation was 56.83. Average disease duration (i.e. time between age at symptom onset and age at death) was 8.67 years based on 12 patients who deceased over the follow-up. Regarding biomarker profiles, all dAD patients were amyloid positive based on CSF and amyloid-PET cut-points, 43/52 were tau-positive based on CSF P-tau or tau-PET, and 4/52 were tau-negative based on CSF P-tau (i.e. borderline ATI), and tau information was unavailable for 4/52 patients. dAD participants who were tau-negative based on CSF P-tau levels were considered to have possible dAD. The only dAD patient who underwent post-mortem examination was assigned an A3B3C3 score (Thal phase 5, Braak stage VI, frequent neuritic plaques), corresponding to a high burden of AD pathology (Hyman et al. 2012). Approximatively 44% of dAD patients carried an *APOE4* allele, which is in line with the observation of a lower proportion of carriers in early-onset atypical AD compared with late-onset amnestic AD (Townley et al. 2020), which has been documented in dAD (Townley et al. 2020).

**Table 1:** Demographics, clinical, biomarker, genetic, and cognitive data.

Demographics and clinical	*n*	
Age at symptom onset (mean, SD)	52	53.44 (5.26)
Age at presentation (mean, SD)	52	56.83 (5.02)
Disease duration (mean, min-max)	12	8.67 (4–13)
Sex at birth (Female, Male)	52	34, 18
Education years (mean, SD)	39	15.15 (2.24)
STMS (median, IQR)	49	24 (15–28)
AD biomarkers and APOE4 status		
CSF only	24	A+ (24), T+ (21), T− (4)
Amyloid PET only	3	A+ (3)
CSF and amyloid PET	5	A+ (5), T+ (3), T− (2)
CSF and amyloid and tau PET	13	A+ (13), T+ (13)
No CSF and amyloid and tau PET	5	A+ (5), T+ (5)
Post-mortem examination only	1	A+ (1), T+ (1)
Amyloid-PET SUVR (median, IQR)	27	2.39 (2.26–2.55)
Tau-PET SUVR (median, IQR)	25	2.06 (1.86–2.62)
CSF amyloid (median, IQR)	43	364.85 (286.65–490.675)
CSF total-tau (median, IQR)	43	499.3 (358.9–836.43)
CSF P-tau (median, IQR)	43	77 (60.13–91.65)
Amyloid/tau index (median, IQR)	43	0.44 (0.265–0.625)
P-tau/Aβ42 ratio	44	0.21 (0.14–0.28)
APOE4 positivity	29	13/29
Cognitive performance (scaled scores)		
Verbal working memory (mean, SD)	31	5.78 (2.61)
Visual working memory (mean, SD)	12	8.27 (4.29)
Trail making test B (mean, SD)	32	2.91 (2.94)
Stroop inhibition (mean, SD)	27	3.96 (2.94)
WCST perseverative errors (mean, SD)	11	6.09 (2.59)
Verbal episodic memory immediate recall (mean, SD)	33	4.17 (2.13)
Verbal episodic memory delayed recall (mean, SD)	33	3.35 (1.89)
Visual episodic memory immediate recall (mean, SD)	25	3.25 (2.49)
Visual episodic memory delayed recall (mean, SD)	24	4.4 (2.38)
Verbal fluency (mean, SD)	35	5.83 (3.23)
Visuospatial (mean, SD)	25	6.52 (2.79)
Visuoconstruction (mean, SD)	32	4.78 (2.82)

CSF amyloid, Total-tau, and P-tau are reported in pg/ml. SD = standard deviation; STMS = Short Test of Mental Status; AD = Alzheimer’s disease; CSF = cerebrospinal fluid; A = amyloid; T = tau; PET = positron emission tomography; SUVR = standard uptake value ratio; IQR = interquartile range; WCST = Wisconsin Card Sorting Test.

### Eigenbrains

**Fig. 1:**
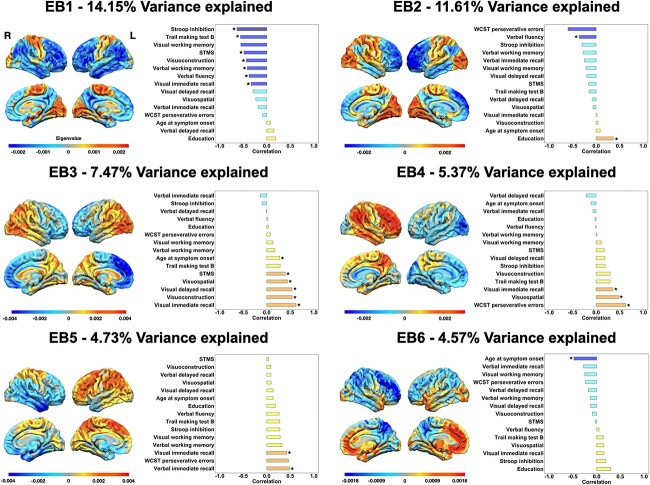
Eigenbrains and their relationships with clinical and cognitive data within the dAD cohort. The color bars represent positive (warm colors) and negative (cold colors) loadings associated with each eigenbrain. These eigenbrains reflect relative metabolism between two sets of brain areas, and the directionality (positive or negative) is arbitrary. Asterisks in the bar plots indicate significant beta coefficients from the multivariable regression analyses. dAD = dysexecutive Alzheimer’s disease; EB = eigenbrain; STMS = Short Test of Mental Status; WCST = Wisconsin Card Sorting Test.

Six eigenbrains were retained for analysis based on Horn’s method. These eigenbrains accounted for a total of 47.89% of covariance in FDG-PET metabolism. Fig. 1 displays these eigenbrains and their relationships with demographic and cognitive data, and Table 2 lists results of the multivariate regression framework.

**Table 2:** Beta coefficients between eigenbrains and clinical/cognitive data.

Variable	*n*	*R^2^*	*R^2^_adj_*	EB1	EB2	EB3	EB4	EB5	EB6	*P*-value
Age at onset	52	0.38	0.30	0.08	0.09	**0.28**	−0.11	0.14	**−0.48**	**<.001**
Education	52	0.29	0.16	0.19	**0.37**	0.03	−0.02	0.18	0.30	0.06
STMS	52	0.46	0.39	**−0.48**	−0.16	**0.39**	0.18	0.04	−0.03	**<0.001**
Verbal WM	31	0.41	0.26	**−0.41**	−0.28	0.17	0.02	0.33	−0.14	**<0.05**
Visual WM	12	0.32	0.00	−0.55	−0.22	0.13	0.11	0.29	−0.25	0.84
Trail making test B	32	0.47	0.34	**−0.56**	−0.15	0.29	0.30	0.28	0.14	**<0.01**
Stroop inhibition	27	0.58	0.44	**−0.63**	−0.30	−0.10	0.19	0.28	0.20	**<0.01**
WCST-P	11	0.61	0.02	−0.09	−0.60	0.08	**0.63**	0.46	−0.24	0.51
Verbal immediate recall	33	0.45	0.32	−0.18	−0.25	−0.13	−0.07	**0.48**	−0.28	**<.05**
Verbal delayed recall	33	0.08	0.00	0.15	−0.08	−0.01	−0.21	0.09	−0.18	0.88
Visual immediate recall	25	0.69	0.59	**−0.33**	0.02	**0.62**	**0.36**	**0.42**	0.16	**<.01**
Visual delayed recall	24	0.44	0.25	−0.29	−0.20	**0.54**	0.19	0.14	−0.13	0.08
Verbal fluency	35	0.34	0.20	**−0.37**	**−0.36**	0.02	−0.01	0.26	0.05	0.06
Visuospatial	25	0.41	0.22	−0.23	−0.07	**0.44**	**0.48**	0.10	0.16	0.10
Visuoconstruction	32	0.50	0.38	**−0.43**	0.04	**0.54**	0.30	0.08	−0.09	**<0.01**

Significant beta coefficients from the multivariable regression analyses are in bold font. EB = eigenbrain; STMS = Short Test of Mental Status; WM = working memory; WCST-P = Wisconsin Card Sorting Test-perseverative errors.

#### Eigenbrain 1

Eigenbrain 1 (EB1) accounted for 14.15% of covariance in patterns of FDG-PET and reflected a gradient of cortical organization with negative loading in heteromodal cortices (frontal, parietal, temporal areas) and positive loading in primary sensory and motor areas, including primary visual cortices. Regression analyses revealed that eigenvalues negatively associated with clinical impairment as assessed with the STMS, most measures of executive functions (inhibition, TMT-B, verbal working memory), visuoconstruction, and visual episodic memory immediate recall. In other words, a patient with a negative eigenvalue would exhibit greater hypometabolism in heteromodal cortices, a lower STMS score, and poorer performance on tasks tapping into executive functioning, visuoconstuction, and visual episodic memory.

#### Eigenbrain 2

Eigenbrain 2 (EB2) accounted for 11.61% of covariance in patterns of FDG-PET and expressed a gradient of cortical organization with negative loading mostly situated in frontal areas and, to a lesser extent, the middle and inferior temporal and supramarginal gyrus with a slight predominance toward the left hemisphere. Positive loading was found in primary sensory and motor areas, including primary visual cortices. Regression analyses showed that eigenvalues negatively associated with verbal fluency and positively associated with years of education. This means that a positive eigenvalue was associated with more hypometabolism in frontal, supramarginal, and inferior temporal areas relative to primary sensory and motor areas, worse performance on verbal fluency tasks, and higher level of education, and vice-versa.

#### Eigenbrain 3

Eigenbrain 3 (EB3) accounted for 7.47% of covariance in patterns of FDG-PET and mainly reflected an anterior-to-posterior gradient of cortical organization with negative loading in prefrontal areas and positive loading in occipito-parietal regions. Regression analyses revealed that eigenvalues positively associated with age at symptom onset and score on the STMS as well as cognitive domains requiring the processing of visual stimuli, including visual episodic memory (immediate and delayed recall), visuocontruction, and visuospatial abilities. In other words, a negative eigenvalue was associated with more hypometabolism in posterior areas relative to frontal regions, an earlier age at symptom onset, and worse performance on the STMS and task tapping into visual processing, and vice-versa.

#### Eigenbrain 4

Eigenbrain 4 (EB4) accounted for 5.37% of covariance in patterns of FDG-PET and reflected a gradient of hemispheric asymmetry with negative loading in the left occipito-temporal areas as well as sparse area throughout the frontal cortex and positive loading in heteromodal cortices of the right hemisphere. Multiple regression analyses revealed that eigenvalues positively associated with cognitive domains requiring the processing of visual stimuli, i.e. visual episodic memory (immediate recall) and visuospatial abilities, as well as the number of perseverative errors on the WCST. This means that a negative eigenvalue was associated with more hypometabolism in the right parieto-frontal network relative to the left hemisphere and worse performance on the cognitive tasks described above, and vice-versa.

#### Eigenbrain 5

Eigenbrain 5 (EB5) accounted for 4.76% of covariance in patterns of FDG-PET and reflected a gradient of hemispheric asymmetry with negative loading in the right prefrontal and parieto-temporal areas with a peak in the temporal pole and positive loading mostly situated in parieto-frontal areas of the left hemisphere. Multiple regression analyses revealed that eigenvalues positively associated measures of verbal and visual episodic memory (immediate recalls). In other words, a negative eigenvalue was associated with more hypometabolism in the left parieto-frontal network relative to the right hemisphere and worse performance on episodic memory recalls, and vice-versa.

#### Eigenbrain 6

Eigenbrain 6 (EB6) accounted for 4.57% of covariance in patterns of FDG-PET and reflected a gradient of cortical organization with negative loading mostly situated in parieto-frontal areas bilaterally with a predominance toward the right hemisphere and positive loading in medial prefrontal and anterior and medial temporal lobes bilaterally with a predominance towards the left hemisphere. Multiple regression analyses revealed that eigenvalues only negatively associated age at symptom onset. This means that a positive eigenvalue was associated with more hypometabolism in the parieto-frontal network relative to fronto-temporal areas and an earlier age at symptom onset.

### *Neurosynth* decoding of eigenbrains

Complete results for the *Neurosynth*-based decoding of eigenbrains can be found in Supplementary Table 1. Relative hypometabolism in heteromodal cortices (EB1), including parietal areas (EB3), was associated with meta-analytic topics of working memory, numerical operations, language perception and semantics and visual attention (negative values), as opposed to topics of sensory and motor abilities (positive values). Interestingly, these eigenbrains did not relate to the topic of response inhibition, a known core executive function. Eigenbrains reflecting anterior-to-posterior gradients (EB2 and EB3) showed relationships between relative hypometabolism of anterior areas and meta-analytic topics associated with so-called “frontal” functions (i.e. moral/social reasoning, decision making) (negative values), reward and negative emotion, and working memory, whereas relative hypometabolism in posterior areas related mostly to topics of mental abilities requiring the processing of visual information (e.g. perception, directed gaze, visual attention) and stimulus response (positive values). Although both EB4 and EB5 are related with to the topic of working memory and response inhibition, relative hypometabolism of the left parieto-frontal network (positive values of EB5) is associated with topics of numerical operations, error learning, language perception and motor, and relative hypometabolism of the right parieto-frontal network (negative values of EB5) associated with topics such as reward, negative emotion, moral reasoning, and facial recognition. EB6 showed associations between relative hypometabolism of medial fronto-temporal areas and topics of moral/social reasoning, reward, decision making and episodic memory (positive values), and relative hypometabolism of parieto-frontal areas and topics of executive functions (working memory, numerical operations), visual abilities (visual attention, perception, directed gaze), and motor (negative values).

### Four dAD subtypes

#### Neuroimaging comparisons

**Fig. 2:**
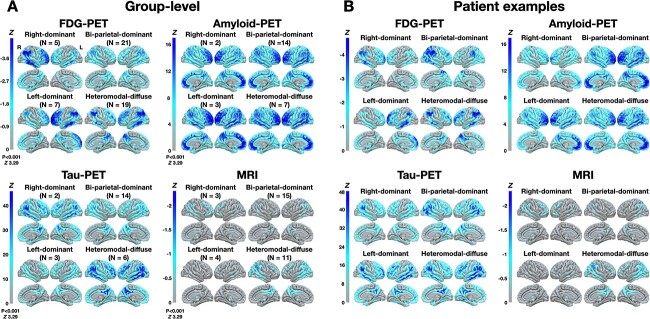
Imaging profiles of dAD subtypes. (A) Group-level analyses of dAD subtypes compared to 52 cognitively unimpaired amyloid-negative controls matched for age and sex, expressed in *Z* scores. (B) Patient-level analyses of “prototypical” dAD patients for each subtype, where each patient was compared to the same 52 controls in a similar manner as group-wise analyses. dAD = dysexecutive Alzheimer’s disease; PET = positron emission tomography; MRI = magnetic resonance imaging.

The hierarchical clustering analysis performed on eigenvalues of the six significant FDG-PET-based eigenbrains revealed four distinct dAD subtypes. Imaging profiles of each subtype are displayed in Fig. 2 (group- and patient-level). Supplementary Fig. 1 shows a heatmap and dendrogram of the clustering. Supplementary Fig. 2 shows a confirmatory analysis comparing dAD subtypes on their eigenvalues for each eigenbrain.

The first subtype is referred to as “right-dominant” (*n* = 5), as it was characterized by hypometabolism in heteromodal cortices of the right hemisphere compared with the control group. The second subtype is referred to as “bi-parietal-dominant” (*n* = 21) and highlighted an overall milder pattern of hypometabolism mostly concentrated in parietal areas bilaterally. The third subtype is referred to as “left-dominant” (*n* = 7) as it was characterized by hypometabolism in heteromodal cortices of the left hemisphere. The fourth subtype is referred as to “heteromodal-diffuse” (*n* = 19) as it showed hypometabolism in heteromodal cortices bilaterally.

Comparisons of dAD subtypes and controls on other imaging modalities revealed spatial patterns of tau-PET uptake similar to those of FDG-PET hypometabolism in each dAD subtype. It is, however, noteworthy that tau-PET hemispheric predominance was less evident in the left-dominant dAD subtype, which could possibly be due to a small sample size (i.e. three participants). A similar observation was seen in patterns of neurodegeneration as assessed with MRI, where patterns of neurodegeneration were similar to those of FDG-PET hypometabolism in each subtype. However, none of the comparisons reached the *P* < 0.001 uncorrected threshold of significance for MRI. Comparisons of amyloid-PET deposition between dAD subtypes and controls highlighted very similar patterns across dAD subtypes, with amyloid deposition mostly concentrated in heteromodal cortices. Given the small sample size for left- and right-dominant dAD groups, we included individual maps for each imaging modality for patient with available multimodal imaging in Supplementary Fig. 3.

#### Clinical and cognitive comparisons

**Fig. 3:**
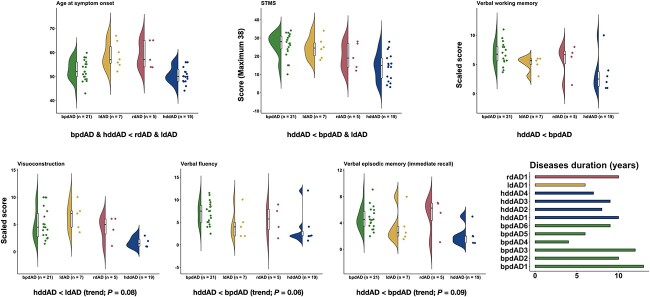
Clinical and cognitive comparisons between dAD subtypes. Significant and trending comparisons between dAD subtypes are reported. The disease duration is displayed for each dAD patient that died over the follow-up. More details about these comparisons are reported in Table 3. dAD = dysexecutive Alzheimer’s disease; STMS = Short Test of Mental Status; bpdAD = bi-parietal-dominant dAD; hddAD = heteromodal-diffuse dAD; ldAD = left-dominant dAD; rdAD = right-dominant dAD.

**Fig. 4:**
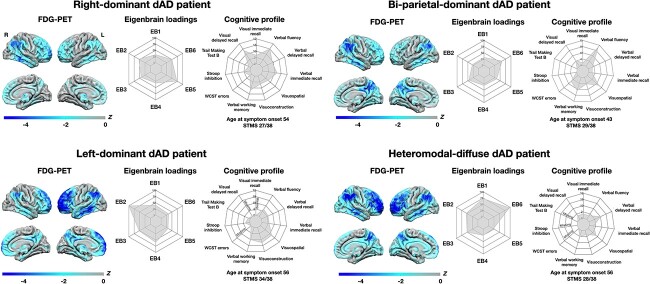
FDG-PET, eigenbrain loadings, and cognitive profiles of individual dAD patients*.* One prototypical patient per dAD subtype with available neuropsychological data is displayed. For each patient, the left portion displays the pattern of FDG-PET hypometabolism compared with 52 cognitively unimpaired amyloid-negative controls, expressed in *Z* scores. The middle portion shows a radar plot with loadings on all six eigenbrains, where a score of 0 means this patient has the lowest eigenvalue on this given eigenbrain across the dAD cohort, a score of 100 indicates this patient has the highest eigenvalue and a score of 50 indicates a median eigenvalue. The right portion displays a radar plot with cognitive scores expressed in scaled scores (ranging from 0 to 12), with age at symptom onset and STMS score below. Neuropsychological tests that were not administered were assigned a score of 0 and are indicated as “missing” inside the plots. dAD = dysexecutive Alzheimer’s disease; PET = positron emission tomography; EB = eigenbrain; STMS = Short Test of Mental Status; WCST = Wisconsin Card Sorting Test.

Comparisons of demographic, clinical and cognitive data between dAD subtypes are summarized in Table 3 and Fig. 3. Additionally, Fig. 4 shows FDG-PET pattern, eigenbrain loadings, and cognitive profiles of four dAD patients (one per subtype). Of note, these case examples were chosen arbitrarily to represent the canonical extremes of phenotypic spectrum of dAD as this facilitates the understanding of these data driven features by presenting the cases that capture the extremes. Subtypes differed at age of symptom onset, where the bi-parietal and heteromodal-diffuse subtypes had a younger age at symptom onset than left- and right-dominant subtypes. Heteromodal-diffuse patients had lower scores on the STMS than bi-parietal and left-dominant patients, and worse verbal working memory than bi-parietal patients. Although only at the trend level, the heteromodal-diffuse subtype exhibited lower scores on measures of verbal fluency and verbal episodic memory compared with the bi-parietal subtype, and poorer performance on measures of visuoconstruction compared to the left-dominant subtype. dAD subtypes did not differ on other demographic or cognitive variable, or in disease duration.

**Table 3:** . Demographic and cognitive comparisons between dAD subtypes.

Variable	*n*	bpdAD	hddAD	ldAD	rdAD	*P*
Sample size	52	21	19	7	5	-
Age at symptom onset	52	52.76 (4.29)	50.74 (3.56)	58.86 (5.46)	59.00 (5.61)	**<0.001** ^**a**^
Education years	52	14.22 (1.90)	15.93 (2.58)	16.75 (0.96)	14.67 (1.15)	0.06
Sex (Female, Male)	52	10, 11	4, 15	2, 5	2, 3	0.30
Disease duration	12	9.5 (3.46)	8.5 (1.29)	6.00^*^	10.00^*^	0.76
STMS	52	26.33 (6.48)	14.24 (7.57)	24.67 (5.92)	20.00 (7.31)	**<0.001** ^**b**^
Verbal working memory	31	6.86 (2.06)	3.50 (3.39)	5.13 (1.26)	5.71 (2.89)^c^	**<0.05** ^**d**^
Visual working memory	12	8.00 (5.48)	8.00^*^	8.00^*^	9.50 (0.71)^c^	0.93
Trail making test B	32	3.53 (3.26)	1.00^c^	2.40 (2.62)	2.50 (3.00) ^†^	0.45
Stroop inhibition	27	5.07 (3.17)	2.67 (2.89)^c^	2.00^*^	2.25 (0.96)^c^	0.14
WCST perseverative errors	11	6.50 (2.27)	-	8.00^*^	3.50 (3.54)^c^	0.28
Verbal episodic memory immediate recall	33	4.66 (1.72)	2.20 (1.64)	3.50 (2.62)	5.12 (2.84)^c^	0.08
Verbal episodic memory delayed recall	33	3.39 (1.92)	3.00 (2.12)	3.00 (1.12)	4.00 (2.74)^c^	0.86
Visual episodic memory immediate recall	25	3.64 (2.90)	1.50 (0.58)^c^	3.33 (1.53)^c^	3.67 (2.52)^c^	0.52
Visual episodic memory delayed recall	24	5.07 (2.02)	2.00 (1.41)^c^	4.33 (3.06)^c^	4.33 (3.51)^c^	0.15
Verbal fluency	35	7.05 (2.55)	3.57 (3.82)	4.60 (3.29)	5.50 (3.39)^c^	0.07
Visuospatial	25	6.56 (2.98)	4.83 (0.24)^c^	7.62 (2.36)^c^	5.94 (3.56)^c^	0.71
Visuoconstruction	32	5.08 (2.85)	1.75 (0.96)^c^	6.50 (2.57)	4.25 (2.36)^c^	0.07

a bpdAD and hddAD < ldAD and rdAD (<.01);

b hddAD < bpdAD (<.001) and ldAD (<0.05);

c Less than five observations were available. dAD = dysexecutive Alzheimer’s disease; ldAD = left-dominant dAD; bpdAD = bi-parietal-dominant dAD; rdAD = right-dominant dAD; hddAD = heteromodal-diffuse dAD; STMS = Short Test of Mental Status. Values are expressed as mean and standard deviation (in parentheses).

d hddAD > bpdAD (<0.05).

e Only one observation was available.

## Discussion

The overarching objective of this investigation was to decipher the clinico-radiological heterogeneity of a relatively large cohort of dAD patients utilizing unsupervised machine learning techniques. The progressive dysexecutive syndrome associated with AD pathology is characterized by a remarkable heterogeneity in the concomitantly observed patterns of FDG-PET hypometabolism. This heterogeneity can be linearly parametrized by a relatively small number of biologically interpretable eigenbrains mainly reflecting heteromodal to primary motor/sensory, left–right hemispheric asymmetry, and anterior-to-posterior gradients of macro-scale cortical organization. Importantly, these eigenbrains captured inter-individual variability in clinical and cognitive impairment, which is supported by both the brain–behavior associations within the dAD cohort and the associations between the eigenbrains and external meta-analytic topics reflecting a wide range of mental abilities. A clustering analysis revealed four data-driven dAD subtypes, which meaningfully differed in their clinical and imaging profiles. These findings have important implications for clinical and therapeutic strategies for patients with dAD and inform the nature of the components of executive functions impaired in dementia syndromes.

### Associations between eigenbrains and cognition

EB1 accounted for the highest proportion of covariance in patterns of FDG-PET metabolism across the dAD cohort and opposed heteromodal cortices to unimodal (i.e. sensorimotor, primary visual) areas. This result fits with previous studies consistently finding a principal gradient of macro-scale cortical organization situating associative and unimodal cortices at opposing ends of its axis (Margulies et al. 2016; Raut et al. 2020; Brown et al. 2022; Jones et al. 2022). Heteromodal cortices identified by this eigenbrain are thought to be the latest to have developed over the course of human evolution (Buckner and DiNicola 2019) and are heavily involved in the modeling of both internal and external data to guide complex forms of behavior and mental representations (Margulies et al. 2016; Jones et al. 2022). This eigenbrain is highly reminiscent of archetypical patterns of degeneration in dAD (Townley et al. 2020; Corriveau-Lecavalier et al. 2022a), and is consistent with previous findings showing that this latent pattern of network degeneration could distinguish dAD from several other dementia syndromes including typical and atypical AD (Jones et al. 2022). Therefore, its relationships with a wide range of high-level cognitive function, especially those tapping into executive functions (both within-cohort and in the *Neurosynth* analysis), were expected. This eigenbrain likely reflects common patterns of network degeneration across dAD patients and may underly the cognitive symptomatology at the core of this syndrome. However, this eigenbrain did not relate to the meta-analytic topic of response inhibition. This suggests that the selective degradation of these areas in dAD might relate to the deterioration of working memory and cognitive flexibility (Jones and Graff-Radford 2021; Uddin 2021) rather than cognitive and behavioral disinhibition. Indeed, this latter symptom is rather a cardinal clinical feature of bvFTD (Rascovsky et al. 2011; Godefroy et al. 2021; Ranasinghe et al. 2021) and is associated with the degeneration of ventromedial prefrontal areas (Jones and Graff-Radford 2021).

The remaining eigenbrains mostly highlighted anterior-to-posterior (EB2 and EB3) and left–right hemispheric asymmetry (EB2, EB4, EB5) gradients of macro-scale cortical organization, and may reflect within-syndrome heterogeneity. EB3 highlighted relationships between relative parietal hypometabolism and cognitive tasks and meta-analytic topics mostly associated with the processing of visual stimuli. This fits with the known involvement of these areas in the processing of visual information and the generation of object-based representations (Swaminathan and Freedman 2012; Xu 2018), also known as the “dorsal visual pathway” (Freud et al. 2016). It is also noteworthy that this eigenbrain was related to clinical impairment as assessed with the STMS, similarly to EB1. These findings underscore the longstanding yet underappreciated involvement of parietal areas in distributed networks subserving executive functions and global cognition (Koenigs et al. 2009; Jones and Graff-Radford 2021; Uddin 2021).

Eigenbrains revealing relative hypometabolism in heteromodal cortices of the left hemisphere (EB2, EB5) generally associated with cognitive tasks of verbal fluency and episodic memory as well as meta-analytic topics relating to language perception and numerical operations. This is in line with the known lateralization of the brain that is relevant to cognition and that has been demonstrated through the decomposition of functional MRI signal (Shine et al. 2021; Brown et al. 2022) and the asymmetric predisposition of network degeneration seen across a wide variety of dementia syndromes (Buciuc et al. 2021; Townley et al. 2021; Jones et al. 2022). This finding is also consistent with studies suggesting that the processing and manipulation of sequential and/or local information, especially (but not restricted to) verbal material, would be tied to the integrity of left hemispheric portion of the parieto-frontal network (Osaka et al. 2003; Osaka et al. 2004; Fiez 2016; Flevaris and Robertson 2016). Of note, the positive association between eigenbrain 2 and education may seem counterintuitive as it indicates that relatively greater hypometabolism in prefrontal areas was associated with worse verbal fluency capacities but higher educational attainment. This may reflect a more abrupt cognitive decline in individuals with higher levels of education once a certain threshold of brain damage is exceeded, as predicted by the cognitive reserve model (Stern 2009; Barulli and Stern 2013) and supported by findings in large longitudinal cohorts of older adults with incident dementia (Wilson et al. 2019). Relative hypometabolism in the heteromodal cortices of the right hemisphere associated with poorer visual episodic memory, visuospatial, and set-shifting (i.e. WCST) performance as demonstrated by within-cohort brain–behavior expressed by EB4, as well as meta-analytic topics of reward, negative emotion, moral reasoning, and facial recognition as expressed by negative values of EB5. The fact that these eigenbrains relates not only with domains requiring the processing of visual information but also with tasks and topics related to cognitive flexibility and social reasoning indicates that the right hemisphere may be involved in the processing of holistic, contextual, and global information (Osaka et al. 2003, 2004; Flevaris and Robertson 2016; Patel et al. 2018), whereas EB3 might be involved in the “pure” processing of visuospatial information, as described above.

EB6 explained the lowest proportion of variance across the set of significant eigenbrains and only related with age at symptom onset. Thus, it is possible that this eigenbrain relates with biological disease properties that are not prominently associated with cognition. It is interesting that the pattern of relative hypometabolism expressed by this eigenbrain resemble to what is seen in limbic-predominant age-related TDP-43 encephalopathy (LATE) (Botha et al. 2018, 2019; Grothe et al. 2022; Tremblay et al. 2011). One possibility is that this eigenbrain might reflect subtle amounts of co-pathologies that may not yet have relevance to cognitive symptomatology, but this remains highly speculative and autopsy data will be required to support this statement.

### Four dAD subtypes

The dAD cohort could be further divided into four subtypes based on an unbiased, data-driven hierarchical clustering algorithm. Group-wise comparisons of FDG-PET images between each subtype and a sample of age- and sex-matched cognitively unimpaired controls revealed a “left-dominant,” a “right-dominant,” a “bi-parietal-dominant,” and a “heteromodal-diffuse” subtypes. Spatial patterns of hypometabolism generally corresponded to those of tau-PET and MRI neurodegeneration in each dAD subtype, which is consistent with a large body of literature showing a spatial correspondence between these modalities as well as their close relationship with cognitive symptomatology (Ossenkoppele et al. 2016; Dronse et al. 2017; Jones et al. 2017; Schöll et al. 2017; La Joie et al. 2020; Rubinski et al. 2020; Franzmeier et al. 2020a, 2020b; Vogel et al. 2021). Conversely, patterns of amyloid-PET did not substantially vary across subtypes, which is again in line with prior findings showing that spatial patterns of amyloid deposition are common across Alzheimer’s disease phenotypes (Jones et al. 2016; Graff et al. 2021). Overall, imaging findings indicate high radiological heterogeneity across dAD subtypes. Although the causes underlying such inter-individual variability remain largely elusive, some studies have suggested that this selective network susceptibility might be related to shared genetic expression (Richiardi et al. 2015; Vértes et al. 2016), cortical microstructure (Huntenburg et al. 2018; Paquola et al. 2019), and regional molecular characteristics (Grothe et al. 2018; Sepulcre et al. 2018). Extrinsic factors may also play a role and interact with these intrinsic factors to heterogeneously alter large-scale spatial neurodynamics (Grün et al. 2022), such as neurodegeneration in dementia, as recently hypothesized (Jones et al. 2022). Future research is needed to explore these avenues.

Several differences were observed when comparing clinical and cognitive features between dAD subtypes. The most striking finding is the overall more severe clinical and cognitive profile associated with the heteromodal-diffuse subtype compared with other subtypes. This is consistent with the more severe and diffuse imaging profile evidenced across imaging modalities (FDG-PET, tau-PET, MRI) in this subtype. The fact that this subtype had a younger or similar age at symptom onset and similar disease duration compared to other subtypes argues that the more severe clinical impairment may not reflect late-stage disease. This aligns with longitudinal cases where executive dysfunction was a first and early finding rather than a late-stage feature (Corriveau-Lecavalier et al. 2022a), and previous studies directly comparing dAD with typical AD showing that the degeneration of heteromodal association cortex was specific to dAD, whereas the degeneration of the medial temporal lobe was specific to typical AD (Townley et al. 2020; Jones et al. 2022). In contrast, the bi-parietal-dominant subtype showed an overall milder cognitive profile despite a younger age at symptom onset, in accordance with our previous findings (Corriveau-Lecavalier et al. 2022a). Left- and right-dominant subtypes did not significantly differ from each other nor from other subtypes on cognitive measures, which is likely due to the small sample sizes. It is however interesting to note that average performance on key tasks, although not significant, was in the expected direction based on our previous clinical case series (Corriveau-Lecavalier et al. 2022a). Indeed, left-dominant dAD patients exhibited, on average, worse performance on measures of verbal capacities (i.e. verbal fluency, verbal episodic memory) compared with bi-parietal-dominant and right-dominant patients, and right-dominant dAD patients had lower visuoconstruction performance compared with left-dominant patients. Moreover, results highlighted by the spectral covariance decomposition analysis support the hypothesis that dAD patients with predominant FDG-PET hypometabolism in the left- or right- hemisphere would be characterized by greater impairment in tasks involving verbal and visual capacities, respectively.

It is noteworthy that the clustering algorithm used in this study yielded a subtyping solution that is highly consistent with the three subtypes previously described in our clinical case series (Corriveau-Lecavalier et al. 2022a). This is particularly interesting, given that methodological approaches between the two investigations radically differ, where our previous study relied on the qualitative description of clinical cases while the analysis used in the present study is purely data-driven. This led to the observation of a new dAD subtype (i.e. heteromodal-diffuse) as well as clinical and imaging differences between subtypes that had not been previously described. Of note, this algorithm accurately classified five of the six cases included in this previous case series (Corriveau-Lecavalier et al. 2021a), with the remaining case being classified as heteromodal-diffuse while it was previously categorized as bi-parietal. This highlights the potential of unsupervised machine learning techniques to uncover brain–behavior relationships relevant for clinical practice and research but that could have otherwise been missed if based on clinical observations alone.

### Executive function components

**Fig. 5:**
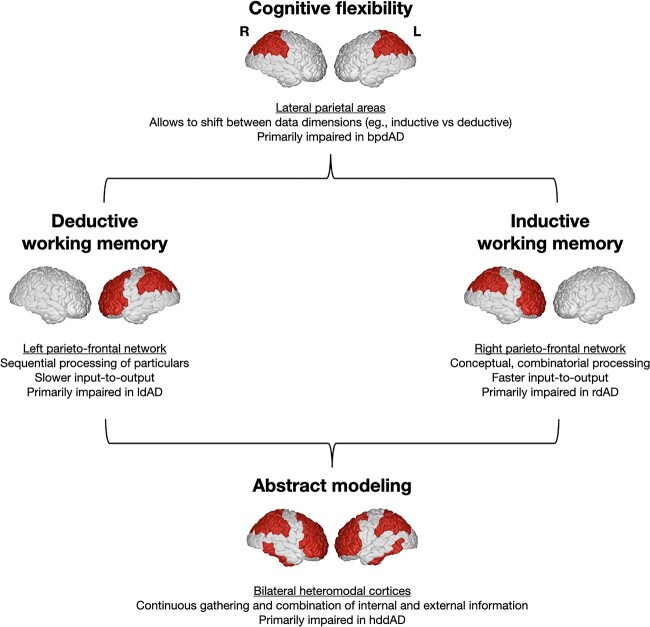
Components of executive functions reflected by patterns of network degeneration in dAD. A proposed conceptual framework of executive functions in degenerative dementia informed by the clinico-radiological associations observed in dAD and the recently proposed global functional state space computational model of mental functional relevant for dementia syndromes in general (Jones et al. 2022). The core, canonical “working memory” is fragmented in the “deductive” and “inductive” components, which are anatomically subserved by the left and right hemispheric portions of the parieto-frontal network, respectively. The deductive working memory is involved in the sequential processing and manipulation of particulars (e.g. words, lists of items) and has a slow processing time. On the other hand, the inductive working memory is involved in the processing and manipulation of global, conceptual and combinatorial information and has a faster processing time. The “cognitive flexibility” component relevant to dAD would be primarily subserved by lateral parietal areas and would allow to shift between data dimensions (i.e. inductive and deductive). Finally, the “abstract modeling” component within a short-term store would be supported by heteromodal cortices and would be involved in abstract mental modeling from the continuous processing and integration of internal and external data sources.

The clinico-radiological heterogeneity of dAD indicates that different patterns of network degeneration are associated with the impairment of variable aspects of executive functions. This led us to formulate a conceptual framework of executive functions relevant to degenerative dementia, which is depicted in Fig. 5. In this model, we propose a fragmentation of the core, canonical working memory into “deductive” and “inductive” components, which are anatomically tied to the left- and right-hemispheric portions of the parieto-frontal network, respectively. Deductive working memory is involved in the sequential processing of particulars and local information and has a slower processing time. An example of this would be the sequential processing of a list of auditory or visual items to retain in mind or a sequence of words forming a sentence. This component would be primarily impaired in left-dominant dAD. Conversely, inductive working memory would be involved in the processing and manipulation of global, combinatorial, and contextual information (i.e. the “gestalt”) and has a faster processing time. Examples of involving the deductive working memory would be the recognition of visual patterns or features of speech requiring the processing of global and contextual information including prosody, humor, and intention. This component would represent the primary impairment in right-dominant dAD.

This “inductive” versus “deductive” working memory components distinction represents the main divergence between our model and Baddeley’s model of working memory (Baddeley and Hitch 1974; Baddeley 1996, 2003, 2010, 2012). This latter model rather divides the canonical working memory into the “phonological loop” and “visuospatial sketchpad” components, which are thought to serve as temporary repositories allowing to maintain and manipulate speech-like and visual information, respectively (Baddeley and Hitch 1974; Baddeley 1996, 2003, 2010, 2012). In our model, the fact that the deductive and inductive working memory components are more heavily involved in the processing of speech-like (or language) and visuospatial information, respectively, is not due to a mere verbal versus visual dichotomy, but rather relates to the particular/local versus global/conceptual processing of incoming data streams. This would explain why degeneration of the left parieto-frontal network in dAD relates to the impairment of both verbal and visual episodic memory tasks (i.e. EB5) and meta-analytic topics such as numerical operations and language perception, since all these mental operations require the sequential processing of stimuli. On the other hand, the fact that the degeneration of the right parieto-frontal network relates to the impairment of visuospatial reasoning and error-monitoring capacities (i.e. WCST) (i.e. EB4) and meta-analytic topics such as reward, negative emotion, and facial recognition (i.e. EB5) would be explained by the fact that these operations require a holistic, global processing of information. Other examples from the literature supporting this claim are studies finding a left/right hemispheric lateralization in the processing of sequential/local versus simultaneous/global features of visual stimuli in both human (Flevaris and Robertson 2016) and animals (Bianki 1983, 1984), and grammatical/semantic versus emotional/prosodic speech information (Mesulam 2016; Patel et al. 2018; Gainotti 2019). Therefore, we consider that Baddeley’s model does not entirely capture brain–behavior associations revealed by our findings and existing literature due to the restrictive nature of the data processing assumed to be performed by its components. We consider that recasting the conceptualization of hemispheric lateralization from “verbal” versus “visual” to “deductive” versus “inductive” better encapsulates the cognitive deficits and macro-scale anatomy associated with dAD and degenerative dysexecutive syndromes in general (Jones 2020; Corriveau-Lecavalier et al. 2022a; 2022b).

Other components from our model have already been well-documented in the literature. For instance, the “cognitive flexibility” component would be subserved by lateral parietal areas (Selemon and Goldman-Rakic 1988; Adcock et al. 2000; Uddin 2021; Corriveau-Lecavalier et al. 2022a), and would allow to shift between data dimensions (e.g. inductive versus deductive). This component would be primarily impaired in bi-parietal-dominant dAD. Finally, the “abstract modeling” component would be associated with bilateral heteromodal cortices and would serve as a global “cache” integrating external input and outputs from working memory components to produce and orient complex behavior and mental operations within a global functional state space (Jones et al. 2022). This is in line with previous studies finding that this gradient of macro-scale physiology is positioned at the end of an unimodal-heteromodal spectrum, is involved in abstract functions (Margulies et al. 2016; Raut et al. 2020; Brown et al. 2022; Jones et al. 2022), and the observation that its impairment distinguishes dAD patients from other degenerative phenotypes (Jones et al. 2022).

Is it important to keep in mind that this framework remains conceptual and calls for future work in populations of aging and degenerative disorders selectively targeting executive functions to better refine our understanding of theses cognitive processes. One particularly informative area to refine our understanding of executive functions would be the development of neuropsychological tests focusing on the predicted differences between these two conceptualizations of the components of working memory in aging and AD.

### Clinical and therapeutic considerations

The fact that varying patterns of network degeneration differentially corresponded with clinical and cognitive impairment in dAD warrants individualized strategies in terms of diagnosis, counseling, and symptom monitoring. When encountering a patient for whom dAD is in the differential or biologically supported, the addition of FDG-PET assessment may help determine the pattern of network degeneration and consequently the most likely dAD subtype. We emphasize the utility of FDG-PET as the patterns of network degeneration were strikingly more prominent and phenotype-specific compared with MRI. This is in line with well-established frameworks suggesting a better sensitivity of FDG-PET to AD-related changes compared to MRI (Sperling et al. 2011; Jack et al. 2013) and our recent work showing that FDG-PET is superior to MRI as an input feature for deep learning algorithms for synthesizing tau-PET images (Lee et al. 2022). However, direct assessment between FDG-PET and MRI to delineate patterns of brain network degeneration and dAD subtypes are pending to support this statement, but this analysis is not within the scope of the present study. The assessment of clinical features (e.g. age at symptom onset) and different component of executive functions that may yield different cognitive profiles (e.g. cognitive flexibility versus working memory processes used in language functions or supporting visual task performance) can also provide useful information for clinical phenotyping and defining more specific features for dAD subtypes. Consequently, information about patterns of network degeneration, in addition to clinical phenotyping, should prompt referral to appropriate resources such as neuropsychology, speech, and language pathology and/or others to determine the nature and extent of cognitive deficits and formulate recommendations accordingly. For instance, symptom monitoring and counseling in the context of a heteromodal-diffuse versus bi-parietal dominant dAD case should differ given the substantial difference in cognitive impairment severity between these two subtypes. Thus, dAD subtyping could help tailoring desired endpoints in clinical trials as suggested elsewhere (Graff et al. 2021), guide the design of non-pharmacological interventions such as cognitive interventions targeting the rehabilitation of specific mental abilities (Belleville et al. 2011, 2014, 2022), and interventions targeting large-scale networks such as transcranial magnetic stimulation (Chard et al. 2021; Koch et al. 2022). This study also emphasizes the importance of stratification by age at symptom onset for various trials (e.g. 50+ versus 60+), given the significant relationship between this age, clinical severity, and phenotypic presentation (Graff et al. 2021).

It is also important to emphasize the relevance of data-driven approaches in general to build disease models with physiology as a starting point rather than clinical observations alone. The very nature of these approaches positions them as promising candidates to unravel the biological features relevant to disease physiology, above and beyond a priori clinical categorizations. For instance, recent studies using unbiased, data-driven techniques have suggested that gradients of macro-scale degeneration relevant to cognition (Brown et al. 2022; Shine et al. 2021) and neurodegeneration (Jones et al. 2022) align with the transcriptomic, myeloarchitectonic and cytoarchitectonic topology of the brain (Burt et al. 2018; Huntenburg et al. 2018) and overlap with neurotransmission systems (Goulas et al. 2021; Hansen et al. 2022). This hints to the possibility that dAD subtypes and dementia syndromes in general might have distinct biological properties leading to selective network degeneration. These techniques inform disease models and advance therapeutic programs aimed at disease biology and consequently targeting and monitoring the effects of such programs.

### Limitations

Our findings must be interpreted in the light of some limitations. Although our sample was deeply characterized at the clinical, cognitive, and imaging levels, it is relatively small. This was particularly evident when it came to comparisons between dAD subtypes, where the sample size for left- and right-dominant groups was considerably smaller than for bi-parietal and heteromodal-diffuse subtypes. Although this may speak to the potential prevalence of these subtypes, it may have considerably hampered our capacity to better characterize their cognitive profile. It is also worth noting that our study was retrospective, and that patient data primarily came from clinical practice. Although this increases generalizability to clinical settings, these aspects also bear the limitation of lack of standardization of neurological and neuropsychological assessments. Moreover, the cross-sectional design of this study precludes the assessment of longitudinal clinical and imaging trajectories, and this is an ongoing line of research in our group. This would allow the determination of patterns of longitudinal degeneration across dAD subtypes. It was not possible to replicate the data-driven clustering in an independent cohort given the lack of FDG-PET data in cohorts of younger onset dAD and the fact that the criteria for a progressive dysexecutive syndrome due to AD have only recently been proposed (Townley et al. 2020). Predicting clinical features and subtype in an independent dAD cohort using this FDG-PET would have been optimal to consolidate the replicability and stability of our findings. However, the clusters were strikingly different in clinical and imaging data not used in the clustering algorithm. Ultimately, these four subtypes may be better viewed as the canonical extremes of a continuous variation along phenotypic dimensions defining a degenerative dementia spectrum (Jones et al. 2022). Finally, although we were able to explain nearly half of the covariance in patterns of FDG-PET hypometabolism, this also means that nearly half of the covariance remains unexplained. Further inquiries probing into variables that may relate to this remaining variance, for instance factors related to brain health (e.g. vascular), cognitive reserve (e.g. cerebral reserve, lifestyle, Stern 2012), or technical factors (e.g. optimized imaging protocols) will be required to further explain this variability.

## Concluding remarks

We leveraged unsupervised machine learning techniques to yield a set of biologically interpretable eigenbrains explaining nearly half of the covariance in patterns of FDG-PET metabolism in a relatively large sample of patients with dAD. Importantly, these eigenbrains captured variability in clinical and cognitive impairment, providing evidence that specific patterns of network degeneration would be tied to clinical and cognitive heterogeneity in this clinical population. Our data-driven hierarchical clustering analysis suggests the presence of four data-driven dAD subtypes, which meaningfully differed in terms of imaging and cognitive profiles. Taken together, these results provide a deeper understanding of the components of executive functions as demonstrated by our framework inspired by the brain–behavior relationships observed in dAD and dementia in general (Jones et al. 2022). Our findings also have important implications for patient care in terms of diagnosis, symptom monitoring and management, and support the use of unsupervised machine learning techniques to gain clinical and biological insights in neurodegenerative dementia syndromes.

## Supplementary Material

Supplemental_Figure1_bhad017

Supplemental_Figure2_bhad017

Supplemental_Figure3_bhad017

Supplementary_Materials_bhad017

## Data Availability

The data supporting these findings of this study can be made available upon reasonable request to the corresponding author.

## CRediT authors statement

Nick Corriveau-Lecavalier (Conceptualization, Data curation, Formal analysis, Methodology, Project administration, Software, Visualization, Writing—original draft, Writing—review and editing), Leland R. Barnard (Conceptualization, Formal analysis, Methodology, Software, Visualization, Writing—review and editing), Jeyeon Lee (Formal analysis, Methodology, Software, Writing—review and editing), Ellen Dicks (Formal analysis, Methodology, Software, Writing—review and editing), Hugo Botha (Conceptualization, Methodology, Supervision, Writing—review and editing), Jonathan Graff-Radford (Conceptualization, Writing—review and editing), Mary Machulda (Conceptualization, Writing—review and editing), Bradley Boeve (Conceptualization, Funding acquisition, Writing—review and editing), David Knopman (Conceptualization, Funding acquisition, Writing—review and editing), Val Lowe (Funding acquisition, Resources, Writing—review and editing), Ronald C Petersen (Funding acquisition, Resources, Supervision, Writing—review and editing), Clifford Jack (Conceptualization, Funding acquisition, Investigation, Methodology, Resources, Supervision, Writing—review and editing), David Thomas Jones (Conceptualization, Formal analysis, Funding acquisition, Investigation, Methodology, Project administration, Resources, Software, Supervision, Writing—original draft, Writing—review and editing).
