# Supplementary figures and images for: Deciphering the clinico-radiological heterogeneity of dysexecutive Alzheimer’s disease
Source: Cereb Cortex. 2023 Jan 31;33(11):7026–43. doi: 10.1093/cercor/bhad017 (PMC10233237; doi:10.1093/cercor/bhad017)

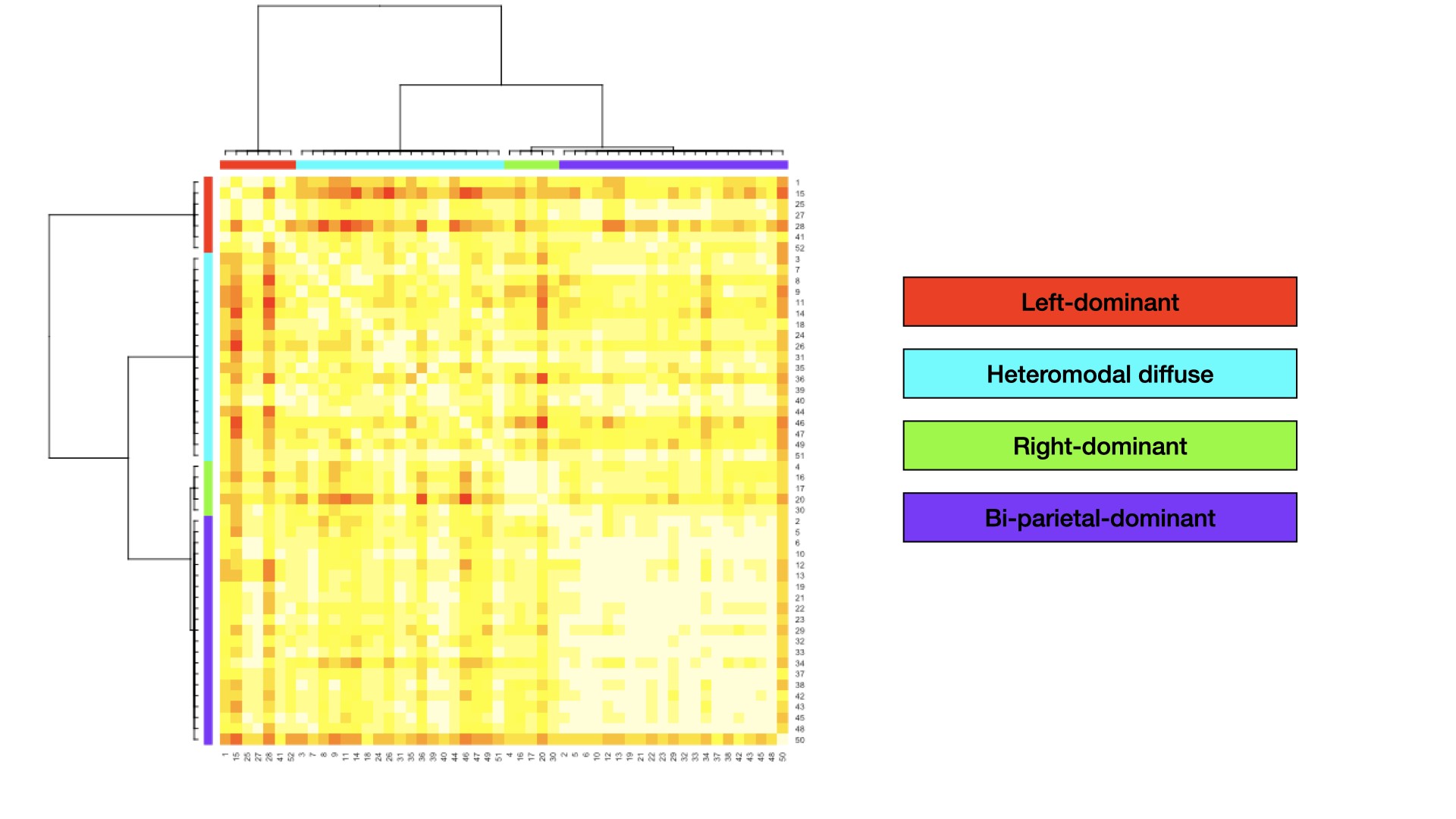

Supplement: Supplemental_Figure1_bhad017 [file supplemental_figure1_bhad017.jpeg]

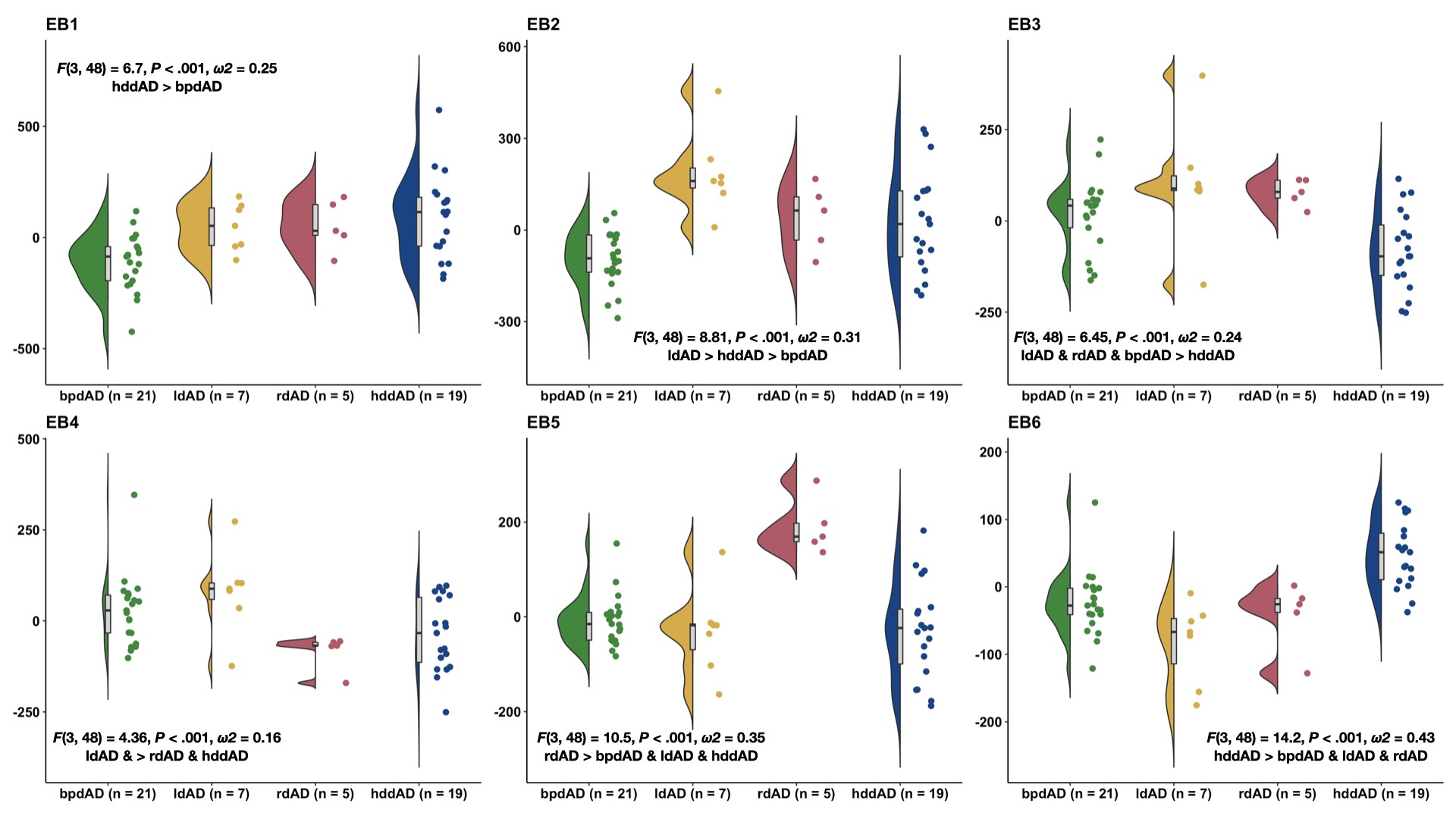

Supplement: Supplemental_Figure2_bhad017 [file supplemental_figure2_bhad017.jpeg]

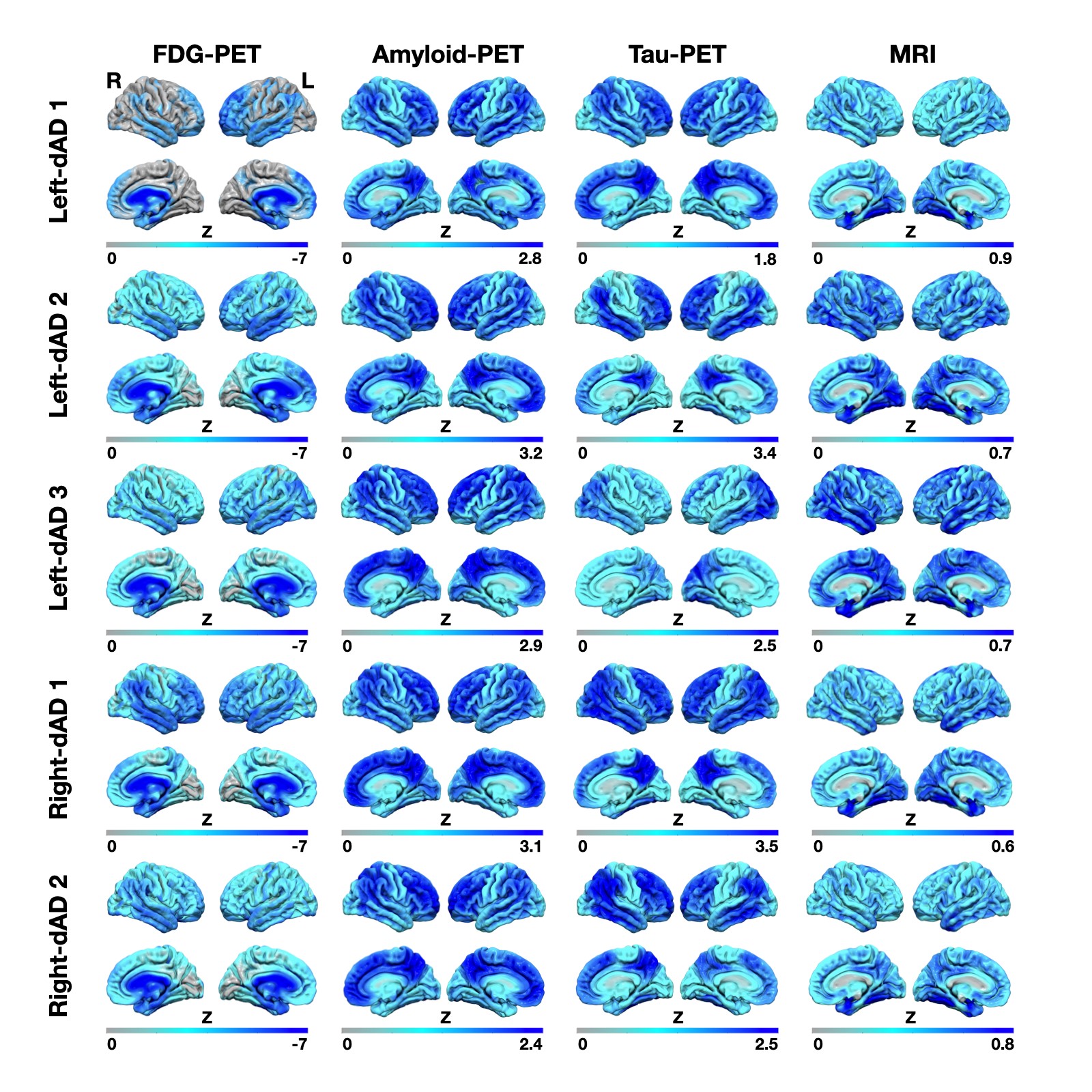

Supplement: Supplemental_Figure3_bhad017 [file supplemental_figure3_bhad017.jpeg]
