# Supplementary material for: Deciphering the clinico-radiological heterogeneity of dysexecutive Alzheimer’s disease
Source: Cereb Cortex. 2023 Jan 31;33(11):7026–43. doi: 10.1093/cercor/bhad017 (PMC10233237; doi:10.1093/cercor/bhad017)
Supplement: Supplementary_Materials_bhad017 [file supplementary_materials_bhad017.docx]

**Supplementary materials**

| Supplementary Table 1 Meta-analytic decoding of eigenbrains using the Neurosynth database | | | | | | | |
| --- | --- | --- | --- | --- | --- | --- | --- |
| Summary term | Neurosynth topic term | EB1 | EB2 | EB3 | EB4 | EB5 | EB6 |
| Stimulus response | 4_stimulus_time_repetition | 0.030 | 0.276 | 0.261 | -0.059 | 0.099 | -0.012 |
| Hearing | 6_auditory_speech_temporal | 0.017 | 0.129 | 0.067 | 0.024 | -0.057 | -0.005 |
| Reward | 7_reward_feedback_striatum | 0.078 | -0.168 | -0.297 | -0.090 | -0.210 | 0.210 |
| Social | 8_mpfc_social_medial | -0.318 | -0.361 | -0.134 | 0.024 | -0.057 | 0.042 |
| Working memory | 9_memory_working_wm | -0.175 | -0.134 | 0.100 | 0.136 | 0.280 | -0.135 |
| Error learning | 11_learning_training_practice | 0.214 | 0.164 | 0.111 | 0.012 | 0.124 | -0.060 |
| Response inhibition | 16_response_inhibition_control | -0.004 | -0.069 | -0.058 | 0.187 | 0.131 | -0.041 |
| Motor | 17_motor_cortex_hand | 0.340 | 0.361 | -0.004 | 0.099 | 0.282 | -0.114 |
| Numerical | 18_number_ips_numerical | -0.210 | -0.058 | 0.271 | 0.060 | 0.229 | -0.115 |
| Negative emotion | 26_emotional_amygdala_negative | -0.017 | -0.207 | -0.232 | -0.081 | -0.340 | 0.217 |
| Moral | 28_social_empathy_moral | -0.205 | -0.332 | -0.230 | 0.045 | -0.216 | 0.112 |
| Decision making | 30_decision_making_risk | -0.111 | -0.314 | -0.265 | 0.010 | -0.057 | 0.113 |
| Pain | 32_pain_somatosensory_stimulation | 0.285 | 0.119 | -0.206 | 0.051 | -0.050 | 0.036 |
| Memory | 33_memory_retrieval_encoding | -0.189 | -0.169 | 0.010 | -0.079 | -0.123 | 0.128 |
| Language perception | 37_language_reading_word | -0.246 | -0.084 | 0.161 | -0.110 | 0.181 | 0.000 |
| Language semantics | 38_semantic_category_representations | -0.295 | 0.095 | 0.261 | -0.080 | 0.049 | 0.007 |
| Facial recognition | 40_face_faces_facial | -0.040 | 0.131 | 0.097 | -0.057 | -0.315 | 0.088 |
| Mental imagery | 41_imagery_mental_events | -0.089 | 0.115 | 0.177 | -0.031 | 0.000 | 0.026 |
| Perception | 42_visual_cortex_sensory | 0.005 | 0.483 | 0.447 | -0.080 | 0.129 | -0.085 |
| Directed gaze | 44_eye_sleep_gaze | 0.205 | 0.360 | 0.199 | 0.027 | 0.186 | -0.111 |
| Motion perception | 45_motion_perception_visual | 0.021 | 0.424 | 0.420 | -0.027 | 0.089 | -0.113 |
| Visual attention | 47_attention_attentional_target | -0.144 | 0.228 | 0.362 | 0.147 | 0.224 | -0.123 |
| Relationships are expressed as correlation coefficients. EB = Eigenbrain. | | | | | | | |

**
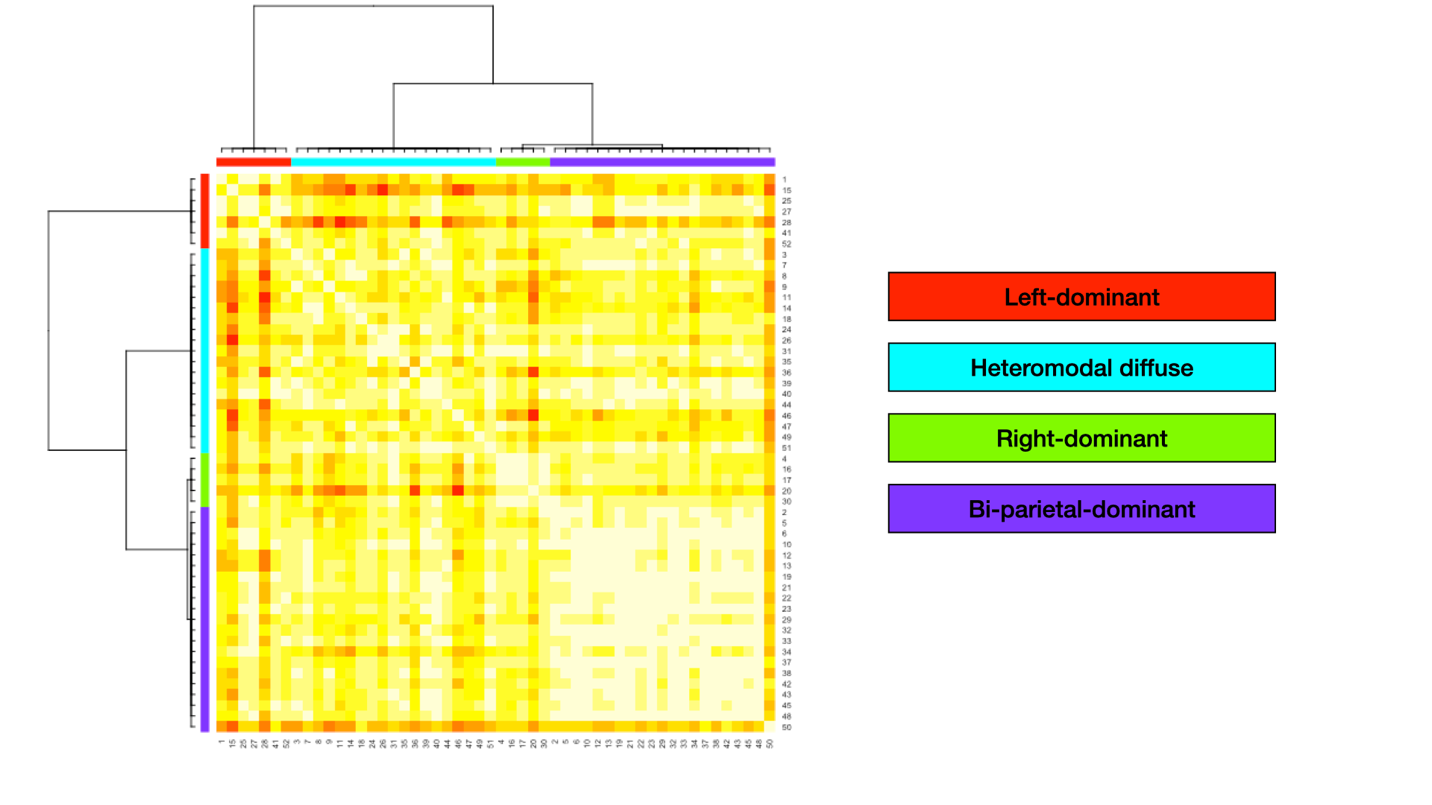
**

*Supplementary Figure 1*. *Heatmap and dendrogram resulting from the hierarchical clustering based on an affinity propagation algorithm*. Each row/column represents the FDG-PET of a single patient based on its eigenvalues on all six significant eigenbrains. Lighter color intensity represents higher similarity between two FDG-PET scans whereas darker colors represent higher dissimilarity. Colors at the borders of the matrix represent clusters, i.e., a dAD subtypes.


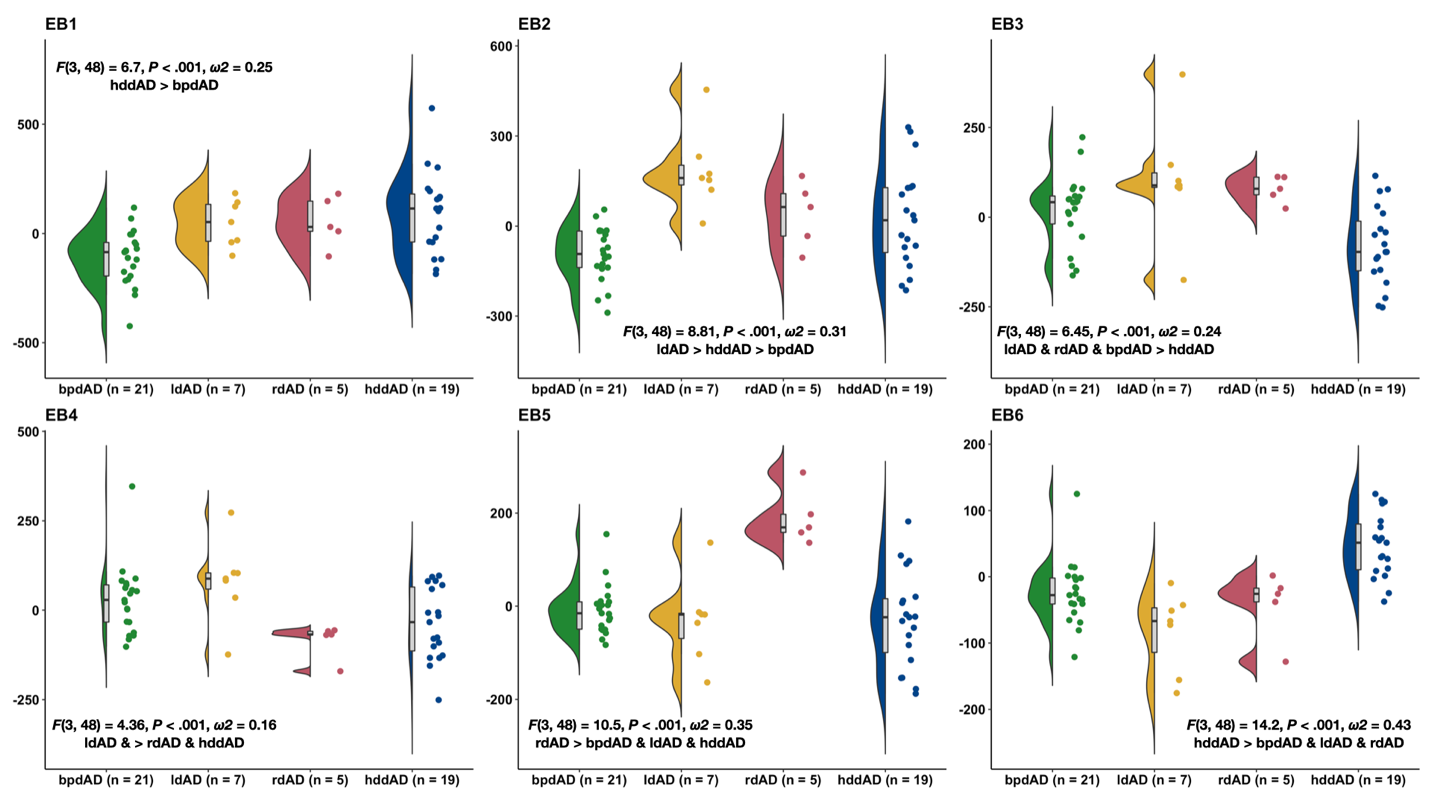


*Supplementary Figure 2*. *Comparisons of dAD subtypes on significant eigenbrains.* Statistical comparisons are reported in the plots. dAD = Dysexecutive Alzheimer’s disease; bpdAD = Bi-parietal-dominant dAD; hddAD = Heteromodal-diffuse dAD; ldAD = Left-dominant dAD; rdAD = Right-dominant dAD.


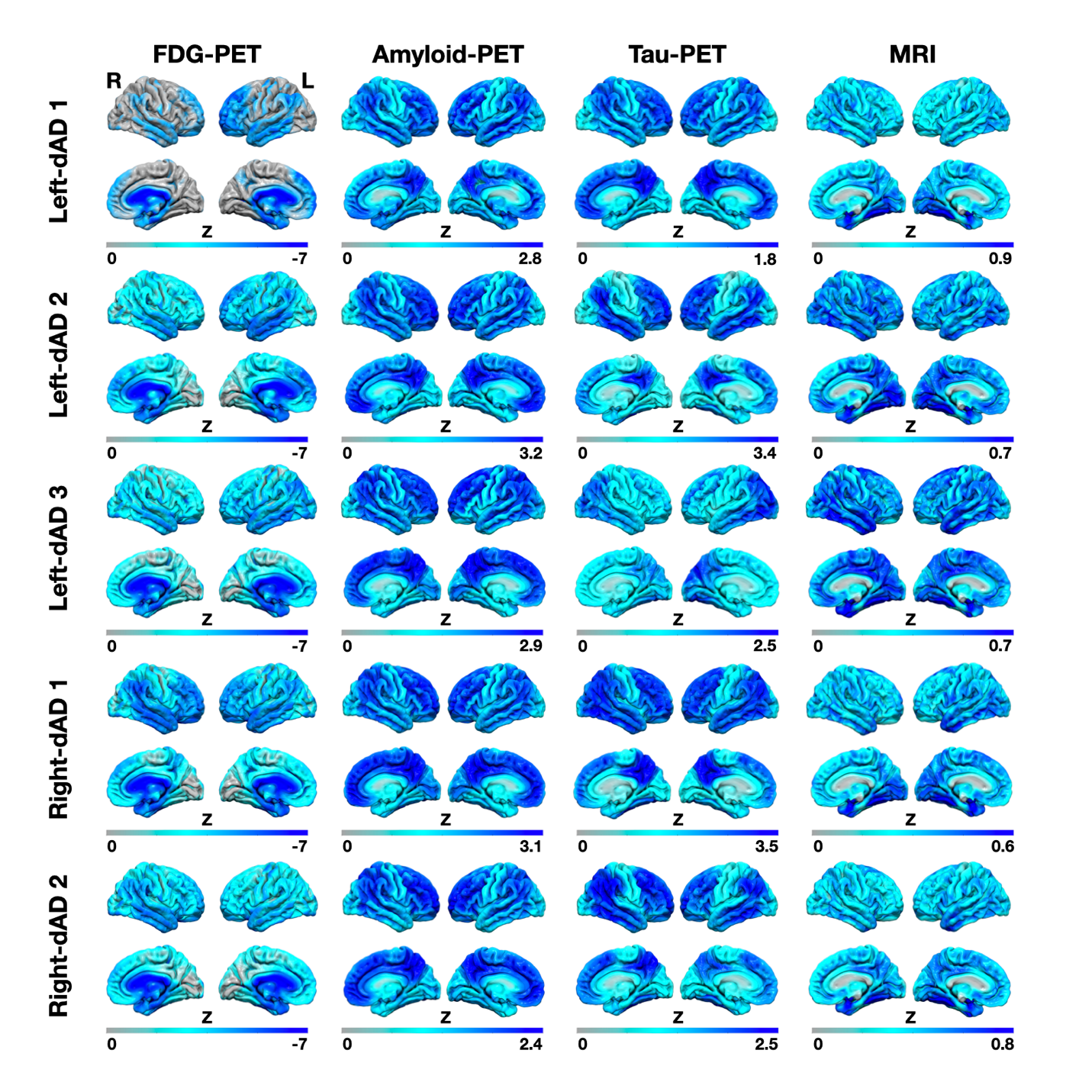


*Supplementary Figure 3*. *Individual images for left- and right-dominant dAD with available multimodal imaging*. Each row is a dAD patient and each column is an imaging modality. Colors express *Z* scores compared to 52 cognitively unimpaired amyloid-negative controls matched for age and sex. dAD = Dysexecutive Alzheimer’s disease; PET = Positron emission tomography; MRI = Magnetic resonance imaging.
